# Bioprocess Strategies for Vitamin B_12_ Production by Microbial Fermentation and Its Market Applications

**DOI:** 10.3390/bioengineering9080365

**Published:** 2022-08-04

**Authors:** Álvaro Calvillo, Teresa Pellicer, Marc Carnicer, Antoni Planas

**Affiliations:** 1 Laboratory of Biochemistry, Institut Químic de Sarrià, University Ramon Llull, 08017 Barcelona, Spain; 2 HealthTech Bio Actives, S.L.U., 08029 Barcelona, Spain

**Keywords:** cobalamin, *Propionibacterium freudenreichii*, *Pseudomonas denitrificans*, cyanocobalamin production

## Abstract

Vitamin B_12_ is a widely used compound in the feed and food, healthcare and medical industries that can only be produced by fermentation because of the complexity of its chemical synthesis. For this reason, finding better producer strains and optimizing their bioprocesses have been the main focus of industrial producers over the last few decades. In this review, we initially provide a historical overview of vitamin B_12_ research and the main biosynthetic characteristics of the two microorganism families typically used for its industrial production: several strains of *Propionibacterium freudenreichii* and strains related to *Pseudomonas denitrificans.* Later, a complete summary of the current state of vitamin B_12_ industrial production as well as the main advances and challenges for improving it is detailed, with a special focus on bioprocess optimization, which aims not only to increase production but also sustainability. In addition, a comprehensive list of the most important and relevant patents for the present industrial strains is provided. Finally, the potential applications of vitamin B_12_ in different markets are discussed.

## 1. Historical Overview

Vitamin B_12_, also known as cobalamin, is a water-soluble molecule essential in many organisms’ metabolism. It has a complex structure and an elaborated biosynthesis, with over 30 biotransformation steps [1]. This biosynthetic pathway is only present in some bacteria and archaea, although the phyla capable of synthetizing vitamin B_12_ are not necessarily interrelated, so mammals, and therefore humans, are unable to synthetize it.

Investigation into vitamin B_12_ began in the 1920s in connection to an illness firstly described in 1824, pernicious anemia. The main symptoms of this illness included fatigue, weight loss, headaches and, in severe cases, dementia, memory loss, muscle weakness and peripheral neuropathy, which can become lethal without treatment. In 1926, Minot and Murphy demonstrated that patients with pernicious anemia could successfully recover from the condition by a special diet with high amounts of lightly cooked liver and muscle meat [2]. They theorized that the treatment was successful because of an unknown “extrinsic factor” present in animal livers. For this discovery, they were awarded the Nobel Prize in Physiology or Medicine in 1934, although it was more than two decades before the so called “extrinsic factor” was identified and isolated. This occurred in 1948, when two research groups from pharmaceutical companies (Folkers at Merck, Sharp & Dohme, and Smith at Glaxo) isolated, almost at the same time, a cobalt compound from animal livers that was able to cure pernicious anemia on its own [3,4]. A year later, the same compound could also be isolated from other sources, such as milk, beef and several bacterial cultures. This red crystalline octahedral cobalt compound was called vitamin B_12_. Interestingly, years later, it was discovered that this compound was in fact one of the many isoforms of the cobalamin (Cbl) family, cyanocobalamin (CNCbl), an artificial physiologically inactive form of cobalamin generated in the industrial process of extraction and isolation from the liver. In addition, CNCbl was the first cobalamin isoform whose structure was solved in 1955 by Dorothy Hodgkin [5].

In 1957, the structure of adenosylcobalamin (AdoCbl), one of the two active forms of cobalamin, was also determined by the same group [5]. These discoveries led to Hodgkin being awarded the Nobel Prize in Chemistry in 1960. Two years later, another physiologically active vitamin B_12_ isoform, methylcobalamin (MetCbl), was discovered. Both AdoCbl and MetCbl were found to act as cofactors in several enzymes and, in the years to come, many MetCbl- and AdoCbl-dependent enzymes were isolated and described. Some of them, such as methionine synthase from *Escherichia coli* and L-methylmalonyl-CoA mutase from *Propionibacterium shermanii*, were crystallized [6,7], as well as most of the molecules responsible for vitamin B_12_ transport in mammals [8,9,10,11,12,13,14,15].

In 1973, after a long study that spanned over a decade, the complete chemical synthesis of vitamin B_12_ was described by Woodward and colleagues [16]. The process was complex, with over 60 steps, including protection and deprotection reactions, and rendered very low yields, less than 1% [16,17].

## 2. Structure of Cobalamin Derivatives and Functions as Enzyme Cofactors

Vitamin B_12_ is the generic name used to designate a family of compounds (cobalamins or Cbl) that share the same common structure: a tetrapyrrolic corrinic ring with a central cobalt atom coordinated to four nitrogen atoms. This scaffold is similar to other prosthetic groups, such as heme in hemoglobin or cytochrome P450. This structure allows the use of the redox state of the central metallic atom, cobalt, allowing the molecule to fulfil its different functions.

**Figure 1:**
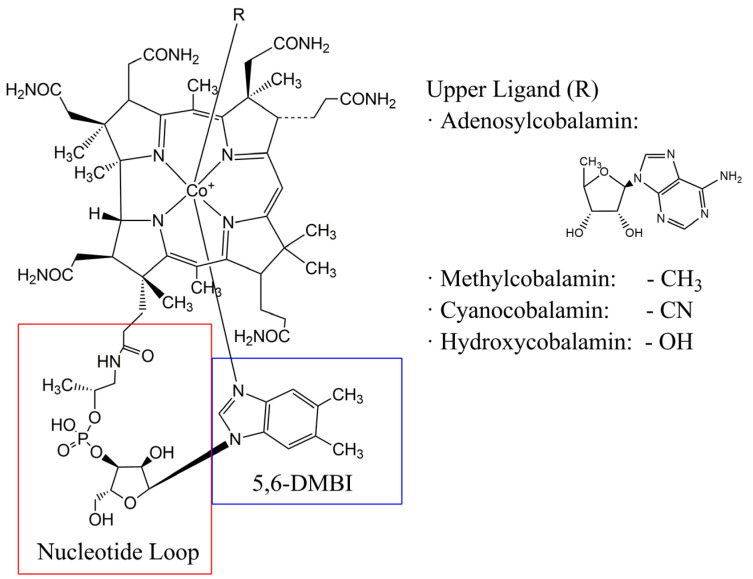
Schematical representation of the structure shared by all cobalamin isoforms. The main groups that usually act as the upper ligand are also shown. 5,6-DMBI: 5,6-dimethylbenzimidazole.

As shown in Figure 1, the corrin ring is formed by four pyrrole units (C_4_H_5_N), joined on opposite sides by a C-CH_3_ methylene link, a C-H link on one side and two pyrrole units directly joined, missing the methine bridge between the A and D subunits present in other known porphyrins, such as hemoglobin. This structure differs from those of other, similar molecules in terms of the number and type of side chains, the oxidation state and the central metallic atom.

Besides the four N atoms of the pyrrole units, the central Co ion is linked to two other ligands. The lower ligand is the base 5,6-dimethylbenzimidazole (DMBI) linked with the central Co ion through the N7-atom in α-axial conformation. DMBI is also linked to one of the side chains of the central corrinic structure: its phosphate group joins to an aminopropanol group that is linked to the propionic acid side chain of the pyrrole unit D of the corrinic ring.

Finally, the sixth ligand is linked to Co in the β-axial position. The nature of this chemical group is variable, presenting different physiological and catalytic functions. For example, a 5-deoxyadenosyl group in this position forms adenosylcobalamin (AdoCbl), while a methyl group forms the methylcobalamin (MetCbl) isoform. In fact, the C-Co found in AdoCbl was the first bond of its type described in a biological molecule [18]. Although these two are the isoforms that present physiological activity as cofactors in humans, other forms, such as hydroxocobalamin (OHCbl) or cyanocobalamin (CNCbl), exist, as well as others with less common upper ligands, such as nitritocobalamin (NitCbl), sulfitocobalamin (SulCbl) or glutathionylcobalamin (GlutCbl) [19].

On one hand, MetCbl is a cofactor of several methyltransferases, such as methionine synthase in humans, an important cellular housekeeping enzyme that functions in two major metabolic pathways: the tetrahydrofolate-dependent one-carbon cycle and the final step in the conversion of methionine from homocysteine. On the other hand, AdoCbl is used as a cofactor by several enzymes, mostly mutases, although only one AdoCbl dependent enzyme is found in mammals: L-methylmalonyl-CoA mutase (MMCM), a critical enzyme for propionate catabolism and degradation of odd-chain fatty acids, several amino acids (valine, isoleucine, methionine, threonine) and cholesterol.

As previously stated, AdoCbl and MetCbl are the active cobalamin isoforms, but they are also known to be light sensitive [20,21]. For this reason, the most common commercial form of vitamin B_12_ is CNCbl instead, which is more stable and readily converted in the body into an active coenzyme form [22].

## 3. Biosynthesis of Vitamin B_12_: The Aerobic and Anaerobic Pathways

The discovery of the structure and biological functions of the different vitamin B_12_ compounds in the 1970s focused the attention of many researchers on the biosynthetic pathways of Cbl-producing organisms. The structural complexity, as it was later established, was due to a large and convoluted biosynthesis that involves more than thirty genes and many enzymatic steps for the “de novo” synthesis of the molecule. This pathway is thought to be exclusive for some bacteria and archaea, as there is no genetic evidence of any eukaryotic organism being capable of producing any isoform of Cbl [1,23].

Although some intermediates were found and isolated earlier [24], it was not until the 1990s that the complete biosynthetic pathway was described in *Pseudomonas denitrificans* [25]. The genes involved in Cbl synthesis were given the prefix cob and a letter that refers to each gene position in the operon. In the following years, the cob enzymes and cobalamin intermediates of *P. denitrificans* were characterized and isolated by the French company Rhône-Poulenc Santé, nowadays Sanofi [26].

Later, genes involved in Cbl biosynthesis were characterized in other organisms, such as *Bacillus megaterium*, *Salmonella enterica* and the previously studied *Propionibacterium freudenreichii*. From the beginning, it was clear that the pathway found in the later organisms was similar to the one found in *P. denitrificans* but genetically different. Key differences included the lack of a monooxygenase and a different cobaltochelatase. Taking this into account, two different pathways for Cbl biosynthesis were established: (i) an aerobic or late cobalt insertion pathway, performed by *P.* *denitrificans* [27] and, as it was later discovered, by other microorganisms, such as *Ensifer casida* and *Sinorhizobium meliloti*; and (ii) an anaerobic or early cobalt insertion pathway, performed mainly by *P. freudenreichii*, *B. megaterium* and *S. enterica* [28].

Independently of the biosynthetic pathway, tetrapyrrole synthesis begins with the synthesis of 5-aminolaevulinic acid (ALA). Thereafter, ALA conversion to a tetrapyrrolic macrocycle structure is performed by three different enzymatic reactions. First, an ALA dehydratase (EC 4.2.1.24), a Zn^2+^ and Mg^2+^-dependent enzyme catalyzes the condensation reaction between two ALA molecules to form porphobilinogen (PBG) [29]. Then, a PBG deamynase (EC 4.3.1.8) polymerizes four molecules of PBG into a linear tetrapyrrole. Finally, a uroporphyrinogen III synthase (EC 4.2.1.75) is able to invert the final pyrrole unit and link it to the first pyrrole unit of the linear tetrapyrrole, forming uroporphyrinogen III, an unsymmetrical hexahydro porphyrin isomer [30]. This molecule is the last intermediate shared with other prosthetic groups, such as heme and chlorophyll groups [31].

**Figure 2:**
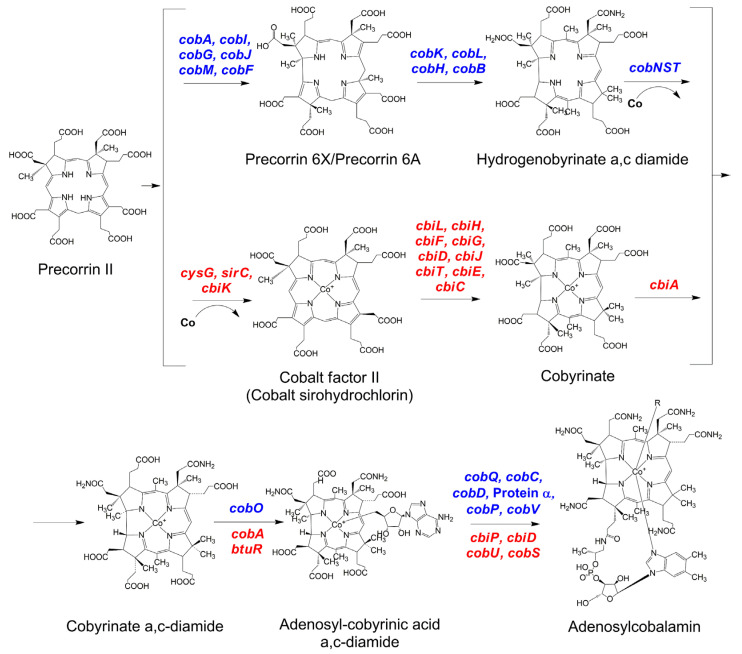
Summary of anaerobic and aerobic adenosylcobalamin biosynthesis. Genes encoding the proteins from the aerobic and anaerobic pathways are shown in blue and red, respectively, except for Protein α whose coding gene is not known.

The transformation of uroporphyrinogen III to precorrin-2, the first molecule in Figure 2, is catalyzed by an uroporhyrinogen III methyltransferase (EC 2.1.1.107), which requires S-adenosyl-L-methione (SAM) as the methyl donor. More specifically, the enzyme methylates at C-2 of the uroporphyrinogen III forming precorrin I and, after a phototrophic tautomerization, the same enzyme is able to methylate at C-7, obtaining precorrin-2, which is the last common intermediate for coenzyme siroheme, P450 and vitamin B_12_ [31,32].

The main differences between the aerobic and anaerobic pathways are in the ring contraction and cobalt chelation steps (see Figure 2). On one hand, the ring contraction in the aerobic pathway requires a molecule of oxygen plus a monooxygenase (CobG) to form precorrin-3B, a hydroxylated γ-lactone intermediate that undergoes a masked pinacol rearrangement during the ring contraction, extruding the methylated C20 position. The ring is then totally contracted, and an acetic acid molecule is liberated in the process [28]. On the other hand, the ring contraction takes place at a later stage in the anaerobic pathway when the cobalt has already been inserted in the molecule. This step is catalyzed by the enzyme codified by the *cbiH* gene, with no molecular oxygen needed. Thereafter, a SAM-dependent methylation takes place at C17, promoting the extrusion of the already methylated C20 position and forming a δ-lactone ring [28]. 

Cobalt chelation is also very different in both pathways. In the aerobic pathway, there is a “late” insertion of the cobalt atom once the ring has been fully contracted. This insertion is catalyzed by an ATP-dependent multienzyme complex (*cobNST*) in the presence of magnesium [28,33]. In the anaerobic pathway, this step takes place at an earlier point on the route, when a cobalt chelatase, encoded by either *cbiX* or *cbiK* genes, catalyzes the cobalt insertion with the ring still in a non-contracted state [28].

In addition, independently of the biosynthetic pathway of the corrin ring, DMBI is produced separately to be later attached in α-axial conformation. The lower ligand synthesis has been described recently and also presents two clearly differentiated routes (the aerobic and the anaerobic pathway), depending on the needs of oxygen.

On one hand, the aerobic biosynthesis of DMBI is catalyzed by the 5,6-dimethylbenzimidazole synthase BluB (EC 1.13.11.79), which performs the fragmentation and contraction of the bound flavin mononucleotide cofactor and the cleavage of the ribityl tail to form DMBI and D-erythrose 4-phosphate in the presence of molecular oxygen. Later, the phosphoribosyltransferase CobU/T (EC 2.4.2.21) introduces the DMBI via a nucleophilic substitution reaction [34]. This pathway was firstly described for *S. meliloti* [35] and later found in the majority of Cbl-producing bacteria [36], including the two most important industrial producers, *P. freudenreichii* [37] and *P. denitrificans* [38]. This fact highlighted the incapacity of *P. freudenreichii* to completely produce Cbl anaerobically without any external addition of DMBI. 

On the other hand, the anaerobic biosynthesis of DMBI is catalyzed by the gene products of the *bzaA-bzaB-cobT-bzaC-bzaD-bzaE* operon, which promote the formation of DMBI with 5-hydroxybenzimidazole, 5-methoxybenzimidazole and 5-methoxy-6-methylbenzimidazole as intermediates. This route was described in the obligate anaerobic bacteria *Eubacterium limosum* [39] and *Acetobacterium woodii* [34].

## 4. Microbial Production of Vitamin B_12_: Bioprocess Optimization for Cyanocobalamin Production

The demand for cobalamin by the food, beverage, dietary and nutraceutical industries has increased sharply in recent years due to the increased health awareness of the general population as well as the growing popularity of alternative diets, such as vegan and vegetarian diets. For this reason, many efforts have been made in strain and process optimization for cyanocobalamin production over the years [1,40].

Besides the cobalamin forms already mentioned, AdoCbl, MetCbl and CNCbl, there are many other cobamides with different lower axial ligands that act as key cofactors for corrinoid-dependent enzymes that are important, for example, for gut microbiota [41,42]. Despite their importance, this review is focused only on cyanocobalamin, the form that can be absorbed and used by humans and which is currently produced industrially.

Historically, the microorganisms used for cobalamin production at industrial scale were strains with high natural productivity, mainly different strains of *P. freudenreichii* and *P. denitrificans*, as well as related strains, such as *Pseudomonas nitroreducens* and *E. casida* [1,40].

For many years, a common strategy to improve these strains has been the usage of random mutagenesis techniques to increase vitamin B_12_ productivity or resistance to toxic intermediates present in media [43]. Nevertheless, overexpression of genes involved in cobalamin biosynthesis [44], heterologous expression of foreign genes [45] and downregulation [46] of several genes have also generated better producer strains. Furthermore, it is worth noting the appearance of new productive strains with promising results, such as *B. megaterium* [47] and *Acetobacter pasteurianus* [48], and the heterologous expression of the biosynthetic pathway in other well-known cell platforms, such as *E. coli*, the later extensively reviewed in Fang et al.’s 2017 study [40]. Recently, Balabanova and co-workers have extensively reviewed the genetic and biosynthetic regulation as well as the genetic tools that have been used with the aim of improving cobalamin production in different cell factories [36].

In contrast, there are also many examples of advances in vitamin B_12_ microbial production by bioprocess optimization. Table 1 summarizes the most relevant innovations performed at a bioprocess level for increased Cbl production and new strategies for Cbl production in new platforms or media. Studies focused on increased production via genetic engineering of known strains are not presented because the objective of this section is to provide an update on the main bioprocess innovations for biotechnological cobalamin production. A summary of culture conditions, media specifications and volumetric productions and productivities is included.

Overall, sharp differences in cobalamin production can be found amongst different producing microorganisms. In this sense, volumetric productions and productivities obtained with *P. denitrificans* are clearly superior to the ones obtained with other producers, while *P. freudenreichii* productions vary widely between strains and culture conditions. In the case of the latter, strategies based on decreasing the propionic acid inhibitory effect seem to be the most effective.

### 4.1. Microbial Production in Pseudomonas denitrificans

*P. denitrificans* is a Gram-negative bacterium that uses the aerobic biosynthetic pathway to produce vitamin B_12_. Despite not having a Generally Recognized as Safe (GRAS) status, *P. denitrificans* is currently the main vitamin B_12_ producer used by industrial manufactures, such as Sanofi in Europe [74] or the Huarong Pharmacy Corporation in China.

On one hand, the Sanofi strain was originally generated by a combination of random mutagenesis and molecular biology techniques and, although no official information about its volumetric production is available, taking into consideration other aerobic strains, it is tempting to speculate that it may produce around 200–300 mg/L [72]. The optimized strain was originated from a natural, high-producing strain known as MB-580, first described and patented in 1962 (US3018225A). Over several years, the French company Rhône-Poulenc amplified several of the cob genes involved in vitamin B_12_ biosynthesis until certain high-producing strains were created—SBL27 and, eventually, SC510 [26]. Sanofi, former Rhône-Poulenc, is now the main European vitamin B_12_ manufacturer.

The dominant worldwide producers of vitamin B_12_ on the market are, however, based in China and include the North China Pharmaceutical Company, the Henan Luyuan Pharmaceutical Company, the Hebei Yuxing Bio-Engineering Company and the Chinese CSPC Huarong Pharmaceutical Company, with a combined reported vitamin B_12_ production in 2020 of approximately 31.41 tons and an estimated value of USD 339.8 million [75]. The origin of the strains used in their industrial productions is not precisely known but assumed to be an aerobic strain due to different publications from research groups related to the Huarong Pharmaceutical Company [69,73,76], and the latest patents on bioprocess optimization with *P. denitrificans* presented in China claim volumetric productions of up to 281 mg/L (see Table 2).

Besides genetic modifications, vitamin B_12_ productivity improvement in *P. denitrificans* has also been achieved with culture media optimization and changes in bioprocessing conditions. For example, the effects of trace elements in media, pH, dissolved oxygen control and the addition of several supplements have been tested. In this sense, the addition of Zn^2+^ has been reported to have a significant positive effect on the synthesis of ALA and PBG, two of the main precursors of cobalamin, while supplementation with Co^2+^ and DMBI, the base that is incorporated into the nucleotide loop, positively affects production [77]. Optimization of the initial amounts of these three compounds by design of experiments led to a 13% increase in cobalamin production [77].

Media composition affected the pH stability of cultures and showed a significant effect on vitamin production. In order to better control pH, a feeding strategy with glucose as carbon source and betaine as methyl donor was developed and was found to be beneficial for vitamin production when applied to 120 m^3^ bioreactor cultures [69,73,76]. Moreover, although it is well known that betaine acts as a methyl donor for vitamin B_12_ biosynthesis [78] and enhances the formation of several key intermediates, such as ALA, glutamate, glycine and methionine [71], high concentrations of betaine can also inhibit cell growth [71,76]. Therefore, a proper betaine feeding strategy was further developed to balance the negative effect on cell growth and the positive effect on cobalamin production and was later successfully implemented at industrial scale [76].

Oxygen transfer rate (OTR) has also been a major subject of bioprocess optimization in *P. denitrificans*. Higher OTRs during initial culture stages enhance cell growth, while lower OTRs in later stages were found to be critical for higher productivity [69]. Later studies revealed that the increased production observed in lower oxygenation conditions can be related to alterations in cell morphology, stimulating change from the cell growth phase to an elongation state that presents higher vitamin B_12_ production [70]. Taking this into consideration, several multi-step dissolved oxygen control strategies were developed, in which aeration and agitation were gradually reduced until dissolved oxygen values fell below 2%, obtaining an improvement in production around 17% [69,73]. Furthermore, the addition of respiratory chain inhibitors, such as rotenone, could also enhance vitamin production despite a detrimental impact on cell growth [67].

Finally, different carbon and nitrogen sources, such as glucose, maltose syrup, beet molasses and corn steep liquor, have been tested as cheaper alternatives to more expensive refined sucrose and glucose. Some of these compounds could negatively affect pH stability and therefore the final vitamin production [68]. Nevertheless, a combination of maltose syrup, corn steep liquor and betaine has been reported as a successful and cheaper alternative to the traditional media compositions [72].

### 4.2. Microbial Production in Propionibacterium freudenreichii

*P. freudenreichii* strains comprise Gram-positive rod-shaped bacteria named after their capacity to synthesize large quantities of propionic acid by the Wood–Werkman pathway. In contrast to aerobic vitamin B_12_ producers, *P. freudenreichii* has the advantage of GRAS status having been granted by the FDA and Qualified Presumption of Safety (QPS) status granted by the EFSA.

Some genetic engineering approaches were tested in *P. freudenreichii* to obtain higher amounts of vitamin B_12_. For example, the overexpression of some of the main genes involved in cobalamin synthesis [44] and a genome shuffling approach [79] were reported to improve cobalamin production. However, the main industrial strains were usually obtained by random mutagenesis using different mutagenic agents, such as UV light or chemical compounds, to obtain better cobalamin producers. In *P. freudenreichii*, these high-yield strains usually present higher tolerance and resistance to propionic acid [43].

*P. freudenreichii* are facultative anaerobic strains that follow the anaerobic biosynthetic route for cobalamin production. Despite their only producing high cobalamin yields at very low-oxygen conditions, oxygen is needed for DMBI synthesis and its attachment to the corrinic ring [80]. For this reason, the culture is usually divided into two stages: a first stage in which the cells are cultured in complete anaerobic conditions and a second stage, usually after 72–96 h of cultivation [59,62,80], in which gentle aeration is provided by agitation to generate the microaeration needed for DMBI synthesis and cobalamin production [74].

The GRAS status of these vitamin B_12_ producers allowed the expansion of their market scope by allowing their direct use in the production of food products. In this sense, in situ food fortification with *P. freudenreichii* has been successfully tested using food-like media, such as in cheese-like propionic medium or whey-based liquid medium [62,64], cereal matrices [63] and in situ fortification of tempeh [81]. Although final cell densities and reported production levels are low compared to other, traditional media, in the context of food fortification, it allows an increase in cobalamin content using non-traditional sources and the achievement of the recommended daily vitamin B_12_ consumption levels with only small amounts of fermented products [64].

As mentioned before, these bacteria have the ability to produce large quantities of propionic acid, which eventually becomes toxic and limits cell growth [52]. Therefore, several bioprocess optimization strategies for decreasing propionic accumulation have been tested. In particular, in situ product removal (ISPR) techniques have shown promising results for the simultaneous production of propionic acid and vitamin B_12_. Among ISPR techniques, the use of expanded-bed adsorption bioreactors (EBABs) with high biocompatibility resins, such as ZGA330, has been reported to support vitamin B_12_ volumetric production levels between 40 mg/L and 60 mg/L [52]. In EBABs, adsorption occurs when the column is expanded, allowing the culture to pass through the chromatographic column without clogging, while propionic acid is retained in the resin [54]. Different culture conditions [54], carbon and nitrogen sources [51] and the addition of media supplements, such as DMBI [53], have been tested for the simultaneous improvement of propionic acid and vitamin B_12_ production. In an EBAB system, the combination of glucose and glycerol [54] and corn stalk hydrosylates [51] have proven to be efficient carbon sources, with reported volumetric CNCbl production levels of 43.2 mg/L and 47.6 mg/L, respectively.

Another interesting approach to decrease propionic acid concentration is the co-fermentation of *P. freudenreichii* with other microorganisms capable of metabolizing propionic acid. For example, the co-culture of *P. freudenreichii* and *Ralstonia eutropha* showed an improved cobalamin production from 6.73 mg/L to almost 19 mg/L [57]. Moreover, co-fermentation has also been successfully applied not only to reduce propionic acid but also to either produce more than one product simultaneously or to fortify other cell cultures. Simultaneous production of both folate and vitamin B_12_ was achieved with the co-cultivation of *P. freudenreichii* and *Lactobacillus plantarum* (currently named *Lactiplantibacillus plantarum* [82]) [55], and a co-fermentation of a *Basidiomycota* strain plus *P. freudenreichii* has been recently patented to simultaneously produce vitamin D and B_12_ [83]. A food fortification example would be the in situ vitamin B_12_ production in bread dough performed in whey-based media with the co-cultivation of *P. freudenreichii* and *Lactobacillus brevis* (currently named *Levilactobacillus brevis* [82]) to ensure microbial safety and stability [56].

Supplementation with cobalamin precursors is another common strategy for increasing productivity. The addition of common precursors and needed compounds, such as ALA and Co^2+^, has often been described as beneficial for vitamin production [1]. Although all *P. freudenreichii* strains are capable of synthetizing DMBI on their own, the biosynthesis of this base is low. Moreover, DMBI formation is not possible in strictly anaerobic conditions, as oxygen is needed for its synthesis [37]. If the availability of DMBI is restricted, the active form of vitamin B_12_ is not formed, and the cells begin to accumulate incomplete forms, such as cobinamide or pseudovitamin B_12_. Thus, the addition of DMBI or even DMBI precursors, such as Riboflavin or Nicotiamide, has been consistently reported as a positive factor in cobalamin production [1,40,53,60,62,64]. In addition, other groups have found that the addition of vitamin B_12_ analogues can decrease feedback inhibition and increase cobalamin production [50].

Finally, *P. freudenreichii* cultures are also interesting in industrial settings for their ability to grow in a wide range of complex carbon and nitrogen sources and even waste and spent media, such as molasses [84], crude glycerol [61], waste frying sunflower oil [58], tomato pomace [85], liquid acid protein residue of soybean [86] and vegetable juice spent media [87].

## 5. Vitamin B_12_ Downstream Processing and Post-Modification Strategies

**Figure 3:**
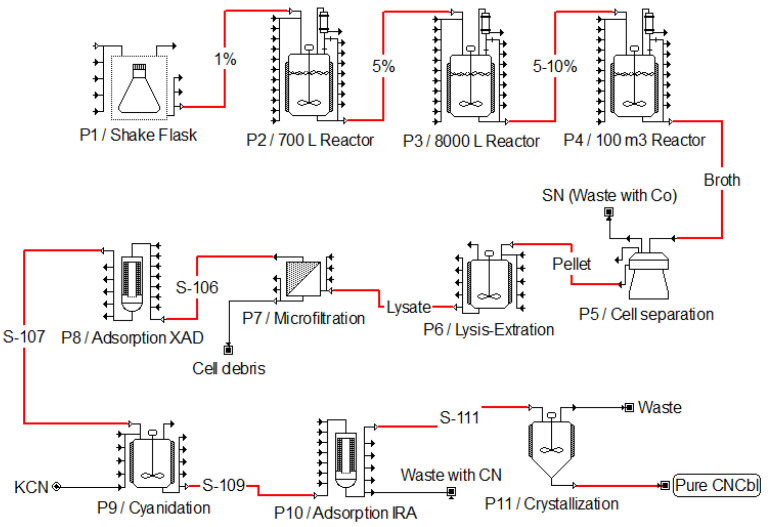
Classical bioprocess to obtain highly pure Cyanocobalamin. The main CNCbl stream is highlighted in red. Intermediate storage vessels are omitted to simplify the figure. P1 to P11 represent Process 1 to Process 11, respectively. Bioprocess represented using SuperPro Designer^®^ V9 Academic Site Edition, Intelligen, Inc. (Scotch Plains, NJ, USA).

Recovery of vitamin B_12_ is a well-described process and, to the best of the authors’ knowledge, has remained unchanged during the past decades at industrial scale (see Figure 3 for a classical bioprocess scheme). Briefly, culture broth is subjected to several separation and purification steps (including extraction, filtration and adsorption processes) which impact on overall process yield and feasibility. Classical downstream processing starts with a biomass concentration to significantly reduce the volume, normally performed by centrifugation. Nevertheless, depending on the bioprocess, Cbl can also be found extracellularly, so purification may start from whole broth.

Either way, all species of corrinoids are extracted by heating at 80–120 °C and a pH of 6.5–8.5 for 10–30 min. Cyanidation can be performed during the extraction process or after the initial filtration and adsorption steps [74,88]. In both cases, the different corrinoids are transformed into CNCbl by the addition of potassium cyanide or thiocyanate. This process is usually performed in the presence of sodium nitrite and heat [80].

Later, CNCbl solution is subsequently clarified by one or more filtration (microfiltration and/or nanofiltration) and adsorption (XAD resin) processes. If the produced Cbl is directed to animal feed, the vitamin solution is often treated with zinc chloride and precipitated with organic solvents, such as acetone, to obtain the final product [89]. When greater purity is required, for example, for pharmaceutical uses, further adsorption steps with different resins (e.g., IRA, Alumina) are often needed to obtain a pure final product. Figure 3 represents a classical bioprocess to obtain highly pure CNCbl.

Once vitamin B_12_ is purified, it may undergo different post-modifications to be used as a food supplement or oral pharmaceutical in order to enhance its bioavailability. Protecting these compounds can be especially interesting in cases of a non-functional intrinsic factor, which causes very low Cbl bioavailability [90,91]. In this sense, several techniques have been developed to protect oral supplements against specific conditions found in the gastrointestinal environment [92]. Among them, microencapsulation, which is already widely used in pharmaceutical and cosmetic industries [93], has proved to improve vitamin B_12_ stability using either food-grade W_1_/O/W_2_ emulsions [94,95], liposomes [96] or different food-grade encapsulating agents, such as chitosan, arabic gum, sodium alginate, carrageenan, maltodextrin, modified starch, cyanobacterial extracellular polymeric, xanthan and pectin [97,98]. Moreover, Fidaleo and co-workers have recently reviewed nanocarrier usage as a promising nanotechnology that may enable vitamin B_12_ therapies to be improved, reducing side effects and overall costs as well as ameliorating the quality of patient lives [99].

## 6. Patents—State of the Art

Research in cobalamin production has been extensively patented since its very beginning, with thousands of patents being published, although most of them are no longer active. Due to the large number of publications and the fact that nowadays most of the production and industrial advances are being made in China, providing a comprehensive list of all the currently active and used patents is difficult and beyond the scope of this review. Instead, Table 2 aims to provide a historical overview of some of the most important and relevant patents for the current industrial strains. We include the state of each patent—as expired, abandoned, or active—in addition to the main innovations claimed and, if available, volumetric production figures.

In 1962, one of the first relevant patents related to the subject after the discovery of the extrinsic factor was US3018225A [100], where the discovery of a natural, high-producing strain (*P. denitrificans* MB580) was described. This strain was extensively researched and many high-producing strains, such as SC510, were obtained through genetic engineering approaches, as described in US20060019352A1 [101]. In fact, researchers associated with Rhône-Poulenc used MB580 and its derived strains to study the genes behind aerobic Cbl biosynthesis and presented the complete aerobic biosynthetic pathway in 1990 [26]. Nowadays, the precise aerobic strains used for industrial cobalamin production are not known but are thought to be closely related to SC510. More recent aerobic strain-related patents cover all stages of bioprocess development: (i) screening and identification of new producer strains (CN111254173 A [102]), (ii) media and bioprocess optimization (CN108949866 A [103], CN110205350 A [104], CN109837320 A [105]) as well as (iii) downstream processing (CN111808158 A [106]).

On the other hand, most of the earliest anaerobic- and *P. freudenreichii*-related patents were focused on strain optimization for CNCbl production. In this sense, one of the most significant early patents is US4544633A [43], where the generation of a propionic-resistant producing strain by random mutagenesis is described. Besides strain enhancement, later patents often focused on bioprocess optimization and the use of bed-expanded bioreactors for the simultaneous production of CNCbl and other compounds of interest, such as propionic acid (US6492141B1 [107]). In addition, the possibility of using Cbl-producing strains of *P. freudenreichii* as probiotics has also been patented (US7427397B2 [108]). Interestingly, the latest patents related to anaerobic strains are focused on either co-cultivation strategies (US9938554 [109], US20200149084A1 [83]) or co-production (CN206828509U [110], IN201827044769 A [111]). The latter patent, IN201827044769 A [111], claims a volumetric production of 76.13 mg/L, which is the maximum production reported for a *P. freudenreichii* strain.

Finally, there are a number of patents with alternative producing strains, such as *B. megaterium* (US2576932A [112]), several *Lactobacillus* strains (WO2011154820A2 [113]), *S. meliloti* (CN104342390 A [114], CN110804598 A [115]) and even *E. coli* (WO2019109975A1 [116]). The production levels of most of these microorganisms are quite low compared to the traditional producers, and patents are often focused on strain identification or strain enhancement by genetic engineering or heterologous expression of the main genes involved in Cbl biosynthesis. However, the exception is the *S. meliloti* strain (CGMCC 9638), which has a vitamin B_12_ production level in the range of 50–115 mg/L [115].

**Table 2:** Main patents related to vitamin B_12_ production.

	Patent Application Number (Reference)	Name	Microorganism/Strain	Innovation	Volumetric Production	Year
*Propionibacterium* genus	US4544633A [43] (Expired)	Process for producing vitamin B_12_ by the fermentation technique, and vitamin B_12_-producing microorganism	*P. freudenreichii* (IFO 12424, IFO 12391, IFO 12426)	Creation of propionic-resistant strains (*P. freudenreichii* FERM-86 and FERM-87) for enhanced CNCbl production	15 mg/L	1983
	US6492141B1 [107] (Expired)	Process for the production of vitamin B_12_	*P. freudenreichii* CBS 929.97	O_2_ effect in production during the anaerobic phase and a "fill and draw" strategy for enhanced production	19 mg/L	1999
	US6187761B1 [117] (Expired)	Production and use of compositions comprising high concentrations of vitamin B_12_ activity	*P. freudenreichii* subsp. *shermanii* and *P. denitrificans*	Method for producing vitamin B_12_ and making highly concentrated compositions	10 mg/L	1999
	US7427397B2 [108] (Expired)	Probiotic *Propionibacterium*	*Propionibacterium jensenii* 702	*Propionibacterium jensenii* as a probiotic	0.0012 mg/L	2004
	EP2376644B1 [118] (Active)	Process for the preparation of a fermentation broth	*Lactobacillus plantarum* DSM 22,118 and *P. freudenreichii* DSM 22120	Fermentation media optimization and co-culture for folate and vitamin B_12_ production	1.07 mg/L	2009
	CN206828509U [110] (Active)	A device for producing propionic acid and co-producing vitamin B_12_ by semi-continuous fermentation	*P. freudenreichii*	Simultaneous production of propionic acid and vitamin B_12_ in a semicontinuous fermentation with propionic acid separation	20.12 mg/L	2017
	US9938554 [109] (Active)	Co-cultivation of *Propionibacterium* and yeast.	*P. freudenreichii* (ATCC 6207) and yeast cells (DSM 28271)	Co-culture of *Propionibacterium* and propionic-resistant yeast to decrease the chemical oxygen load (COD) of spent media	16 mg/L	2018
	US20200149084A1 [83] (Active)	Sequential co-culturing method for producing a vitamin- and protein-rich food product	*Basidiomycota* and *P. freudenreichii*	Co-culture of *Basidiomycota* genus strains and vitamin B_12_-producing strains for in situ food fortification	0.0014 mg/L ^1^	2020
	IN201827044769 A [111] (Active)	Continuous process for co-production of vitamin B_12_ and organic acids	*P. freudenreichii* (ATCC 13673)	Co-production of vitamin B_12_ and organic acids in a continuous fermentation with a single bioreactor	76.13 mg/L	2020
	WO21041759 A1 [119] (Active)	Modified *Propionibacterium* and methods of use	*P. freudenreichii* (P. UF 1)	Generation of a vitamin B_12_-overproducing strain by introducing a mutation that decreases the activity of the cbiMcbl riboswitch	n.d. ^2^	2021
*Pseudomonas denitrificans*	US3018225A [100] (Expired)	Production of vitamin B_12_	*P. denitrificans* MB-580	A process for vitamin B_12_ production with a high-yield strain (*P. denitrificans* MB-580)	2.4 mg/L ^1^	1962
	US20060019352A1 [101] (Abandoned)	Methods for increasing the production of cobalamins using cob gene expression	*P. denitrificans*	Overexpression of several genes involved in Cob biosynthesis; generation of several overproducing strains, such as SC-510	65 mg/L	1990
	US6156545A [120] (Expired)	Biosynthesis method enabling the preparation of cobalamins	*P. denitrificans* G2650	Enhanced Cob production by the heterologous overexpression of precursors, such as DMBI and O-phospo-L-threonine	7.9 mg/L	1996
	CN101538599A [121] (Active)	Method for improving the yield of denitrified pseudomonas vitamin B_12_	*P. denitrificans J741*	Enhance cob production by betaine addition optimization	177.49 mg/L	2008
	CN102399845A [122] (Active)	Vitamin B_12_ fermentation production control process based on CO_2_ concentration in tail gas	*P. denitrificans* MB-580	Vitamin B_12_ enhanced production through a carbon dioxide control strategy during fermentation	164.6 mg/L	2010
	CN101748177 A [123] (Active)	Optimized method for producing vitamin B_12_ through *P. denitrificans* fermentation and synthetic medium	*P. denitrificans*	Development and optimization of media and bioprocess conditions for improved vitamin B_12_ production	77 mg/L	2010
	CN102021214 A [124] (Active)	Oxygen consumption rate-based vitamin B_12_ fermentation production control process	*P. denitrificans*	Vitamin B_12_ production optimization through an oxygen control strategy	171,4 mg/L	2011
	CN102453740 A [125] (Active)	Culture medium for producing vitamin B_12_ by fermenting *P. denitrificans* and fermentation method thereof	*P. denitrificans*	Use of artificial molasses and bioprocess optimization for a more stable fermentation yield	198 mg/L	2012
	CN108949866 A [103] (Active)	Multi-stage rotating speed regulating policy for improving *P. denitrificans* fermentation for production of vitamin B_12_	*P. denitrificans*	Vitamin B_12_ production improved by optimization of the culture media and the stirring speed of the bioprocess	246 mg/L ^1^	2018
	CN108913739 A [126] (Active)	Method for producing vitamin B_12_ by using *P. denitrificans* based on pH value control	*P. denitrificans*	Improved vitamin B_12_ production by optimization of the bioprocess through pH value control	248 mg/L	2018
	CN110205350 A [104] (Active)	Method for improving the yield of vitamin B_12_ based on the regulation of ammonia nitrogen index	*P. denitrificans*	A method for improved Cbl production by supplementation with yeast extract controlled by the ammonia nitrogen index	167 mg/L ^1^	2019
	CN109837320 A [105] (Active)	Method for promoting *P. denitrificans* to generate vitamin B_12_	*P. denitrificans*	Optimization of media and culture conditions for improved vitamin B_12_ production	198 mg/L	2019
	CN111808158 A [106] (Active)	Preparation method of vitamin B_12_ crude product	*P. denitrificans*	Downstream process improvement for AdoCbl extraction	n.d. ^2^	2020
	CN111254173 A [102] (Active)	Screening method and screening culture medium for bacterial strains for high yield of vitamin B_12_ produced through fermentation production with *P. denitrificans*	Several high-yield strains of *P. denitrificans*	Screening for high-vitamin B_12_ producing *P. denitrificans* strains and culture medium screening for high vitamin B_12_ production	281 mg/L ^1^	2020
Other producers	US2650896A [127] (Expired)	Cyanide ions in production of vitamin B_12_	*Streptomyces griseus*	Effects of cyanide ions in B_12_ production	Biological assay	1953
	US2576932A [112] (expired)	Fermentation process to produce vitamin B_12_	*B. megaterium* B-938	Vitamin B_12_ production with *B. megaterium* in a nutrient media with sucrose	0.45 mg/L	1983
	US20050227332A1 [128] (Expired)	Method for producing vitamin B_12_ from hydrogen-metabolizing methane bacterium	A mesophilic methane bacterium obtained from digested sludge	The culture is acclimatized in a H2–CO media and grown in an immobilized bed bioreactor	25.2 mg/L	2005
	US20060105432A1 [129] (Abandoned)	Method for the production of vitamin B_12_	*B. megaterium* DSMZ509	Genetically modified *B. megaterium* strain	0.008 mg/L ^1^	2006
	WO2011154820A2 [113] (Application granted)	Vitamin B_12_-producing probiotic bacterial strains	*Lactobacillus reuteri* (DSM 17938, DSM 16143, ATCC 55730)	In situ food fortification for increased vitamin B_12_ production with *Lactobacillus reuteri* strains	0.018 mg/L ^1^	2011
	CN104342390 A [114] (Active)	*Sinorhizobium meliloti* strain and composition and application of *Sinorhizobium meliloti* strain	*S. meliloti* (CGMCC 9638)	A *S. melitolli* strain capable of producing vitamin B_12_ and optimization of the bioprocess for vitamin B_12_ production	At least 50 mg/L	2015
	WO2019109975A1 [116] (Active)	Recombinant strain of *Escherichia coli* for de novo synthesis of vitamin B_12_, construction method therefor and application thereof	*E. coli*	Recombinant *E. coli* for the de novo synthesis of vitamin B_12_	89 µg/g DCW	2019
	CN110804598 A [115] (Active)	Procorrin-2C(20)-methyltransferase mutant and mutant gene and application thereof in preparing vitamin B_12_	*Sinorhizobium* (CGMCC 9638)	Generation of a vitamin B_12_ overproducer strain by overexpressing the precorrin-2C(20)-methyltransferase gene	115 mg/L	2020

^1^ Values were converted to mg/L using the data available from the original publication; ^2^ n.d.: not determined.

## 7. Vitamin B_12_ Market Applications and the State of the Market

The most important market for B_12_ products is the feed and food industry, where its efficiency and security has been extensively verified [130], although its usage is also extensive in the supplement and pharmaceutical industry. 

In the feed and food industry, CNCbl is commonly added to poultry, pig and calf feeds at dosage levels between 10 to 30 mg/t in almost all Europe and the USA [74]. It is also used as an additive in several food products, for example, in cereal, where its organoleptic properties and chemical properties, such as odorlessness, tastelessness and solubility in water, are an advantage for the fortification of several products. Nevertheless, its bright red color can present a challenge to its addition in other foods, such as white bread [88].

Regarding its usage in the supplement industry, vitamin B_12_ has been gaining relevance in later years, especially with the rise in the popularity of vegetarian and vegan diets [131]. CNCbl is the most used form mainly because of its stability, price, proven safety [22] and its similar efficiency compared to other forms [22,131]. Although there have been some reports of dried algae that contain a significant amount of B_12_ [132], Cbl is virtually absent in vegetables [131], and the main dietary sources are foods derived from animal products. Although B_12_ is present in dairy products and eggs (products suitable for vegetarian diets), their quantities are quite low compared with other options (approximately 0.4 μg/100 g in milk and 1.3 µg/100 g in eggs vs. 9.4 μg/100 g in some meats, 8.9 μg/100 g in fish and 52.4 μg/100 in shellfish [132]). This fact, together with the assumed bioavailability of only 50% of all the Cbl obtained from food sources [41] and the losses that can occur during food processing (cooking, exposure to light, pasteurization, etc.) [131], make reaching the recommended daily dietary intake of 2.4 µg a difficult task for those on pure vegetarian diets without vitamin B_12_ supplements [131].

Vitamin B_12_ deficiency is also prevalent in low- or middle-income countries with mainly plant-based diets and low meat consumption [133]. Even in high-income countries, though, there are several population groups at high risk of B_12_ deficiency. This specially affects the elderly, with around 20% of people over 60 suffering from it in the USA and in the UK according to the NIH [134]. In the case of the elderly, deficiency is mostly due to lower intake and a high prevalence of food-bound malabsorption, caused by age-related gastric atrophy and lower IF levels [41,135].

Other high-risk populations are pregnant and lactating women, children, and patients with autoimmune diseases that cause gastric complications, such as atrophic gastritis or decreased stomach acid secretion [132]. In all these cases, a higher daily B_12_ intake, mainly obtained through supplementation, is recommended. 

Although most supplementations are based on CNCbl, in rare cases of cellular trafficking and protein processing alterations caused by rare genetic diseases [136], supplementation with other forms, such as MetCbl or OHCbl, may be required. Additionally, CNCbl supplementation may be unsuitable for supplementation in smoker populations [137,138].

Finally, it should also be noted that it is often preferred to supplement B_12_ on its own and not as a component of multivitamin tablets because the presence of vitamin C and copper can degrade it and form inactive Cbl by-products [131].

Besides direct supplementation, B_12_ is also widely used for the fortification of different food products. In this case, CNCbl is again the preferred form due to its higher stability when processed and cooked [132]. B_12_-fortified products are common in the United States and other countries where, for example, B_12_-fortified cereals and milk provide a significant amount of the total daily Cbl requirement [132]. Other alternatives, such as flour fortification, have also been considered [139]. In this sense, some of the in situ fortification approaches collected in this review [56,63] may become interesting and valuable alternatives in the future.

Vitamin B_12_ is also widely used in the pharmacological sector, where, besides CNCbl, other forms, such as OHCbl, AdoCbl and MetCbl, are also produced and distributed due to their higher uptake and more sustained serum levels [74]. Pharmacological B_12_ is presented in different forms, such as nasal sprays, oral and sublingual products, and even direct injections to treat pernicious anemia, B_12_ deficiency, cyanide poisoning and lower homocysteine levels. There are also several claims that have been made regarding its positive effect in patients with Alzheimer’s disease and as a stimulant of the immune system, though more evidence is needed to prove these [74,140]. 

Considering all these different usages and markets, it should not be a surprise that vitamin B_12_ total worldwide production and market volume have been steadily increasing, although the exact worldwide market values are difficult to obtain due to the scarcity of reliable information. However, it is safe to assume a great increase in overall production in the last decades. In 1989, the overall production was around 3 tons per year [74] and, by 2005, it had already increased to 10 tons and had a market value of approximately EUR 77 million [88]. As mentioned before, production in China in 2020 reached 31.41 tons with a market value of USD 339.48 million [76], while some projections have been made that the vitamin B_12_ market will reach a total value of USD 410 million by 2027 [141]. The progressive increase in the size of elderly populations, the rise of alternative vegan and vegetarian diets and the scarcity of animal food products are factors that explain this sharp market increase and are also the reasons why the B_12_ market is expected to continue to grow in the future.

## 8. Concluding Remarks

From published data, it is clear that industrial vitamin B_12_ production with *Propionibacterium freudenreichii* strains presents several challenges and shortcomings that must be surpassed in order for this method to compete against those using aerobic strains. From all the reviewed examples (Table 1 and Table 2), the highest volumetric production with the anaerobic strain was 76 mg/L (IN201827044769 A [111]), clearly inferior to the 250–280 mg/L reported for aerobic strains in different studies (CN108949866 A [103], CN108913739 A [126], CN111254173 A [102]).

However, the singularities of markets in which vitamin B_12_ is targeted, such as dietary supplements or fortified foods and drinks, need to be taken into consideration. Many final vitamin B_12_ consumers, besides patients affected by pernicious anemia or other diseases, are vegans or vegetarians and people with high health and ecological awareness. In this scenario, the GRAS status of *Propionibacterium freudenreichii* and the fact that many producing strains are non-GMO microorganisms are valuable assets that increase its market appeal. For example, in situ fortification strategies can become a future economically viable application for *Propionibacterium freudenreichii* cultures. This possibility is further reinforced by the promising probiotic properties described for some *Propionibacterium freudenreichii* strains: microbiota modulation, immunomodulation and the production of several nutraceutical compounds, such as trehalose, naphotic acid and short-chain fatty acids [142].

Moreover, aside from these differentiating traits that can increase the added value for end consumers, the ability of *Propionibacterium freudenreichii* to synthetize different products besides vitamin B_12_ (mainly propionic acid and low amounts of trehalose) can also increase its industrial appeal. Currently, most of the worldwide propionic acid production is obtained from crude oil through petrochemical processes and there have been numerous studies that have aimed to find a bio-based alternative production process that would allow this product to be labeled as a “natural preservative” [143]. Therefore, the possibility of a *Propionibacterium freudenreichii* biorefinery, with simultaneous production and extraction of both propionic acid and vitamin B_12_, would increase the industrial and commercial feasibility.

Besides *Pseudomonas denitrificans* and *Propionibacterium freudenreichii* strains, other possible producers, such as *Sinorhizobium meliloti*, different *Lactobacillus* strains and even *E. coli* (by heterologous expression of the biosynthetic pathway), have also been widely studied. So far, their reported productions are not competitive, making them unsuitable alternatives for industrial CNCbl production. Nevertheless, future strains and strategies may render better production processes which would improve their industrial viability, although their commercial success may be hindered by regulatory constraints and consumer acceptability.

Finally, it should be mentioned that, in its current state, CNCbl production is still suboptimal and has many challenges to overcome to further develop its potential as a cost-effective and valuable industrial bioprocess. The main obstacle is that, even in the higher-producing aerobic strains, such as *Pseudomonas denitrificans*, volumetric production levels are often around 200–300 mg/L—much lower than those obtained via similar fermentation processes, such as those for vitamin B_2_. In addition, the fermentation cycles are long and costly, mainly because of the need for expensive media compounds, such as high concentrations of complex nitrogen sources and supplements such as betaine. Supplying enough cobalt to the broth can also be problematic from a cost and an environmental perspective [144].

Further efforts in bioprocessing, downstream and media composition optimization (with cheaper or recycled compounds) should be carried out to increase the economic viability and environmental sustainability of vitamin B_12_ biotechnological production. However, the main problem, still, is the low productivity of the available producing strains, caused mainly by the tight genetic regulation of Cbl production: the inhibition of the cysG and the cbi operon by the cobalamin riboswitch, as well as other downregulating processes [40,74,144]. Overcoming this limitation may require genetic engineering, which may not be well received by end consumers, mainly vegans or vegetarians, who are very concerned about their diet choices and the usage of GMO organisms.

## Figures and Tables

**Table 1:** Summary of reported productions with the industrial cobalamin producing strains.

Microorganism/Strain	Main Media Components	Scale	Summary/Innovation	Volumetric Production	Volumetric Productivity (mg/L/h)	Reference
*B. megaterium* DSM 319	Terrific broth media	250 mL shake flask	Precursor supplementation and pO_2_ control	0.21 mg/L ^c^	0.006 mg/L/h ^c^	[47]
*Lactobacillus reuteri* ZJ03	Soymilk	250 mL shake flask	Different carbon source supplementation	0.204 mg/L	0.003 mg/L/h	[49]
*P. freudenreichii* subsp. *shermanii* NRRL-B-4327, 3523 and NRRL-B-3524	Sodium lactate broth	250 mL shake flask	Vitamin B_12_ analogue addition	31 mg/L	0.51 mg/L	[50]
*P. freudenreichii* CICC 10019	Glucose, CSL ^a^	7 L stirred tankbioreactor	Expanded-bed bioreactor (EBAB) with crop stark hydrolysates	47.6 mg/L	0.18 mg/L/h	[51]
*P. freudenreichii* CICC 10019	Glucose, CSL	7 L stirred tank bioreactor	EBAB bioreactor	43.4 mg/L	0.27 mg/L/h	[52]
*P. freudenreichii* CICC 10019	Glucose, CSL	1.5 L stirred tank bioreactor	EBAB bioreactor and DMBI addition	58.8 mg/L	0.59 mg/L/h	[53]
*P. freudenreichii* CICC 10019	Glucose/glycerol, CSL	5 L stirred tank bioreactor	EBAB bioreactor, glycerol as carbon source and crop stalk hydrolysate as nitrogen source	43 mg/L	0.36 mg/L/h	[54]
*P. freudenreichii* DF13	Supplemented whey permeate	1 L stirred tank bioreactor	Co-culture with *Lactobacillus plantarum* SM39 for simultaneous folate and Cbl production	0.75 mg/L	0.004 mg/L/h	[55]
*P. freudenreichii* DSM 20271//*Lactobacillus brevis* ATCC 14869	Wheat bran dough	n.d. ^b^	Co-fermentation in wheat bran dough for in situ production of Vitamin B_12_	332 ng/g ^c^	n.d. ^b^	[56]
*P. freudenreichii* IFO 12424//*Ralstonia eutropha* H16 (ATCC17699)	Polypeptone, casein, yeast extract	5 L stirred tank bioreactor	Cell recycling system and co-culture with *Ralstonia eutropha* for decreasing propionic acid inhibition	8 mg/L ^c^	0.14 mg/L/h ^c^	[57]
*P. freudenreichii* PTCC 1674.	Tryptone, yeast extract, different carbon sources	100 cm^3^	Waste frying sun oil as a carbon source for vitamin B_12_ production	2.74 mg/L	0.02 mg/L/h	[58]
*P. freudenreichii* subsp. *shermanii* ATCC 13673	Glucose, yeast extract	2 L stirred tank bioreactor	Inoculum volume, pH control and substrate concentration optimization	0.087 mg/L	0.002 mg/L/h	[59]
*P. freudenreichii* subsp. *shermanii* CICC 10019	Glucose, CSL	100 L fermenter	Addition of DMBI precisely with Ado-Cbl control strategy	39.15 mg/L	0.32 mg/L/h	[60]
*P. freudenreichii* subsp. *shermanii*	Glycerol, tryptone, casein, DMBI	200 mL shake flask	Media optimization by design of experiments with crude glycerol as the main carbon source	4.01 mg/L	0.024 mg/L/h	[61]
*P. freudenreichii* subsp. *shermanii*	Whey based media	20 mL tubes	DMBI, Nicotinamide and Riboflavin supplementation	5.3 mg/L	0.03 mg/L/h	[62]
*P. freudenreichii* subsp. *shermanii*	Food-like media (cereal matrices)	n.d. ^b^	Precursor supplementation in different cereal-like matrices	1.5 mg/Kg	0.009 mg/Kg/h	[63]
*P. freudenreichii* subsp. *shermanii* 2067	Cheese-based propionic media/whey-based liquid media	50 mL shake flask	Production in food-like conditions without DMBI addition	0.124 mg/L ^c^	0.0013 mg/L/h	[64]
*P. freudenreichii* CICC10019	Glucose, yeast extract, CSL	100 mL flasks	Media optimization by statistical analysis	8.32 mg/L	0.068 mg/L/h	[65]
*P. freudenreichii* CICC10019	Glucose, CSL	7 L fermenter	Membrane separation-coupled fed-batch fermentation	21.6 mg/L	0.16 mg/L/h	[66]
*P. denitrificans*	Maltose, peptone, betaine	250 mL shake flask	Addition of rotenone as a respiration inhibitor for enhanced production	54.7 mg/L	0.57 mg/L/h	[67]
*P. denitrificans*	Beet molasses, sucrose, betaine	120 m^3^ fermenter	Glucose-betaine feeding, pH control strategy	214.13 mg/L ^c^	1.27 mg/L/h ^c^	[68]
*P. denitrificans*	Glucose, CSL, betaine	120 m^3^ fermenter	Stepwise oxygen uptake rate control strategy	188 mg/L	1.12 mg/L/h	[69]
*P. denitrificans*	Glucose, CSL, betaine	50 L fermenter	Effects of specific oxygen consumption rate on cell morphology and production	213.1 mg/L	1.88 mg/L/h	[70]
*P. denitrificans*	Maltose, peptone, betaine	250 mL shake flask	Betaine supplementation	58.61 mg/L	0.48 mg/L/h	[71]
*P. denitrificans*	Maltose syrup, CSL, betaine	120 m^3^ fermenter	Maltose syrup and CSL as the main substrates	198.27 mg/L	1.10 mg/L/h	[72]
*P. denitrificans*	Glucose, CSL, betaine	120 m^3^ fermenter	pO_2_ stepwise control	198.80 mg/L	1.18 mg/L/h	[73]

The main microorganism, strain, scale and media compounds are shown as well as a brief summary of the main innovation and the volumetric productions. Volumetric productions are presented in mg/L. Volumetric productivities were calculated using data from the original publications. ^a^ CSL: corn steep liquor; ^b^ n.d.: not determined; ^c^ Values were converted to mg/L or mg/L/h using the data available from the original publication.

## Data Availability

Not applicable.
